# To What Extent are Food, Culture, Economics and the Natural Environment Reflected in the Language of the Australian and Brazilian Food-based Dietary Guidelines?

**DOI:** 10.1007/s13668-024-00585-1

**Published:** 2024-10-07

**Authors:** Natasha Hanssen Midjord, Colin Bell

**Affiliations:** 1 Department of Global Nutrition and Health, VIA University College, Aarhus N, Denmark; 2 Global Centre for Preventive Health and Nutrition, Institute for Health Transformation, Deakin University, 75 Pigdons Road, Geelong, Australia

**Keywords:** Food based dietary guidelines, Australia, Brazil, Language use, Food choice

## Abstract

**Purpose of the Review:**

Our aim was to review literature describing language use in dietary guidelines and explore the extent to which food, culture, economics and the natural environment are reflected in the language of the Australian, compared to the Brazilian food-based dietary guidelines (FBDGs).

**Recent Findings:**

Australia’s FBDGs are based on the best available scientific evidence and claim to “form a bridge between research and evidence-based advice to address the major health challenge of improving Australians’ eating patterns”. Brazil’s FBDGs recognise reasons beyond health for people’s food choices.

**Summary:**

Not a lot of attention has been paid to language use in dietary guidelines. The reviewed studies suggest that language in dietary guidelines should be unambiguous for consumers and evolve with national nutrition priorities. A notable difference between Australian and Brazilian FBDGs was that Australia centralised individuals and individual food groups, whereas Brazil placed people in an ecosystem. Inclusion of words that speak to how food is prepared and eaten, to expressions of culture and community, and to strategies people use for enhancing and protecting livelihoods and planetary health may enhance the relevance of future dietary guidelines.

## Introduction

The third and latest edition of the Australian Food-based Dietary Guidelines (FBDGs) were released in 2013 and target the general healthy population. The guidelines recognise the burden of diet-related non-communicable disease in Australia and claim to “cut through the background noise of ubiquitous dietary advice” to help Australians improve their health [1]. They are based on “the best available scientific evidence” and, “form a bridge between research and evidence-based advice to address the major health challenge of improving Australians’ eating patterns” [1].

Increases in the production, availability, and marketing of ultra-processed foods (UPF’s) and the growing burden of diet related disease over the last 10 years suggests the guidelines may have missed the mark for food producers and consumers [2–4]. A 2011/2012 cross-sectional study found that Australian diets contained more UPFs than minimally processed foods, with 42% of daily energy intake coming from UPF’s [5]. Furthermore, overweight and obesity increased from 57% in 1995 to 67% in 2018 for Australian adults, while coronary heart disease remains the leading cause of death for men and the second leading cause of death for women [6].

With a view to the bridge from evidence to health spanning cultural, economic and environmental reasons for consumer food choices, our aim was to review literature describing language use in dietary guidelines and explore the extent to which food, culture, economics and the natural environment are reflected in the language of the Australian compared to the Brazilian FBDGs [7]. The Brazilian guidelines were chosen as the basis for comparison because of a ‘revolutionary’ focus on people and the planet [8].

## Language Use in Food-based Dietary Guidelines

A brief international literature search was conducted with a focus on Australia and Brazil. We excluded studies focused on children, pregnant or breastfeeding women, or those living with disease, along with studies published prior to 2013.

Box 1: Insights from studies exploring language in food-based dietary guidelines (listed alphabetically by first author surname).

**Table:** 

Author/year	Aim(s)	Study design/ language analysis type	Population	Key findings	Reported Limitations
Anastasiou K et al. 2022	Analyse the terms used and definitions given to ‘Foods to Limit’ in FBDGs around the world in order to provide greater clarity and guidance about the types of terms that can be adopted in future FBDGs	Review	Guidelines from 86 countries	• Past and present FBDGs mainly use nutrient-based terms but food examples and processing related terms have increased in use over the last 20 years • The type of evidence used in dietary guidelines effects its messages → Evidence from randomised controlled trials (*which measures effects of isolated nutrients*) often prioritised over evidence from cross-sectional studies (*which measure effects of foods or dietary patterns*) • ‘Foods to limit’ recommendations are often softened with words like “moderate”, which may be poorly understood by the public • Chosen terms and definitions can be influenced by the worldviews of those involved in development of FBDGs	Unable to access 10 FBDGs provided by FAO due to issues with translation, outdated websites, or lack of public access Only ‘headline messages’ were extracted during data extraction. Terms used in subsequent text were not considered Not all terms were captured due to inconsistency Study was limited to analysing foods to limit due to challenges relating to their terminologies and definitions
Bekele T et al. 2023	Test the acceptability, cultural appropriateness, consumers' understanding, and practicality of the Ethiopian food-based dietary guideline's messages, tips, and food graphics	Qualitative study design	Four included participant groups: 15 high-level nutrition experts, 30 frontline community health extension workers, 15 agriculture extension workers, and 40 consumers	• 11 public messages. First 7 encourage consumers to implement healthier dietary practices habitually. Last 4 public messages advise limiting certain food groups to stay healthy • Acceptability: need for more practical examples of physical activities and cooking demonstrations • Cultural appropriateness: Advice on socially acceptable norms that determine eating behaviour, such as traditional and religious celebrations and socialising, may be useful in influencing healthy food choices. DGs would be more culturally appropriate if they are translated into various local languages (same for graphics) • High-level experts suggested changes to the wording of the messages, particularly on the key messages related to meat consumption and alcohol intake • Consumer understanding: Technical terms (i.e. balanced diet) require a simple definition	This study sample may not represent the entire population Due to subjective nature of qualitative research methods, researchers shape the interactions with participants and their understanding of data, so they may not always reach the same conclusions
Brito de Oliveira M et al. 2022	Evaluate culinary content of key messages contained in FBDGs available on the global online repository of the FAO of the UN	Data were analysed inductively using thematic analysis	73 FBDGs worldwide	• 53.4% included at least one recommendation about cooking in their key messages • When included, most frequent recommendations were those with restrictive content that focused on limiting fat and/or salt during food preparation • Only one FBDG analysed highlighted cooking as a pleasurable practice → valuing and considering expanded concept of health • Only two presented messages about importance of sharing activities related to cooking within the household • The absence of key messages regarding sustainable cooking practices is also an important indicator that this subject is not considered strategic to mitigate climate impacts – a position different from that indicated by recent scientific evidence	Only FBDGs available in the FAO repository were included 22 documents were omitted due to being written in languages not chosen for analysis in this study
Da Silva Oliveira M et al. 2017	Aimed to analyse the advances and limits established in the second edition of the Brazilian FBDG, contrasting with its first edition	Discourse analysis	DGBP 2006 and 2014	• Academics co-authored the 2014 DGs, whereas the 2006 guidelines were authored by the Ministry of Health Graphics: • 2006—reinforce scientific arguments that guided principles and guidelines to convince readers of their rigour • 2014 – Display nourishment realistically. Photos demonstrate important sociocultural aspects of eating Food classification system: • 2006—Foods organised in seven groups based on food nutrient sources → seeks to form basis of dietary and nutritional guidelines aimed at preventing malnutrition, deficiencies, and NCDs by referring to appropriate quantitative consumption of each group • 2014—Foods organised into four groups based on NOVA food classification → justified by impact on diets caused by growing expansion in consumption of UPFs • 2014 recommendations highlight an important distinction between processed foods and ultra-processed foods. The guideline recommends that processed foods may be part of the diet, as ingredients for food preparations or as parts of meals based on in natura or minimally processed foods • The growing scientific and academic knowledge about the unbalanced nutritional composition of UPFs, and their sociocultural and environmental impacts, expresses that there are no longer reasons to recommend ‘moderation’ in consumption of such foods; so they should be avoided/excluded from the diet, in order to make nourishment more appropriate and healthy • Principles for the guideline’s development have changed	Lacks population understanding of the recommendations to analyse strengths and limitations more accurately
Finley J et al. 2017	This article addressed some of the major constraints, challenges, and bottlenecks facing the food system, primarily in the United States	Review	US food system	• Large-scale shifts toward healthier dietary patterns could exacerbate sustainability concerns • Proposes that the first step is for nutrition and public health specialists to establish effective and meaningful discourse with their counterparts in the agriculture and food sectors • Other than providing information to regulatory bodies, nutrition and health professionals have relatively little voice in agriculture and food policy • Economic models show substantial increase in prices of staple foods in recent decades • Some data suggests climate change is affecting nutritional composition of crops • Movement of population toward consumption of healthier diets will require major changes in amounts and types of foods consumed. Such changes in diet could have substantial effects on sustainability of agricultural production and food systems more broadly	
Koios D et al. 2022	Systematically analysed whether and how UPFs are represented in dietary guidelines internationally	Global analysis of consumer-targeted key messages in dietary guidelines	106 dietary guidelines worldwide	• 99% of guidelines utilise some type of nutrient-based messages, either promoting ‘positive’ nutrients or discouraging consumption of ‘negative’ nutrients • Explicit references to food processing were present in 45% of ‘eat less’ guidelines and 5% of ‘eat more’ guidelines • 53% of the specific foods referred to in ‘eat less’ advice were UPFs • Food based advice can reinforce or confuse messages about UPFs; to avoid confusion, policy makers can ensure that ‘eat less’ messages are focused on UPFs • Guidelines could be strengthened by explaining the different levels of processing more clearly	Analysis of full text of detailed guidelines was not possible due to time and resource constraints. As such, authors may have missed some discussion of UPFs Visual food guides were excluded Non-English and non-German documents were excluded
Reyneke G et al. 2022	Aimed to understand Australian consumer perspectives of wording utilised in FBDGs with a focus on legumes and whole grains, in order to evaluate potential suggested wording for future FBDGs	Exploratory study	314 Australian adults (18–65 +)	• Only 28% of Australians consume legumes according to the guidelines • Guidelines need to identify barriers → poor familiarity, lack of prep skills, gastrointestinal discomfort, etc • Consumer confusion around categorisation and quantification (for both legumes and whole grains) • Consumer study found need for more definitive recommendations for whole grain intake • Majority of participants preferred legumes to be featured in only one category • Most participants did not like current wording of whole grain recommendations → most preferred the suggested new statement “choose whole grain products over refined grains/white flour products whenever you can”	Relied on online surveys and may be prone to selection bias Current findings relate to a small female dominant sample of respondents, reporting a baseline legume and whole grain intake well above national average consumption. Therefore, not representative of Australian population Sustainability and environmental factors were not considered part of messaging Could have been strengthened by contribution of communication experts to effectively interpret evidence into messaging appropriate for a range of literacy levels and thereby optimise nutrition communication strategies
Seed B 2015	Outlined recommendations and utilises an ecological framework of policy analysis to examine context, drivers, consequences and future suggestions in establishing and maintaining sustainability principles within the Qatar Dietary Guidelines	Policy analysis	Population of Qatar	• One of the key initiatives in the plan proposes ‘by transitioning to high-efficiency production and better crop selection, farms in Qatar are able to produce approximately 40% of the nation’s food (by volume) using the same amount of land and one-third less water than is required today’ • Recommendations specific to sustainability under the title “Eat Healthy while Protecting the Environment” • Emphasise a plant-based diet, including vegetables, fruit, whole grain cereals, legumes, nuts, and seeds • Reduce leftovers and waste • When available, consume locally and regionally produced foods • Choose fresh, home-made foods over highly processed foods and fast foods • Conserve water in food preparation • Follow the recommendations of the Qatar Dietary Guidelines • ‘Tips’ to carry out the recommendations allude back to other advice on the guideline, stating: ‘Try not to over consume food and drinks. This uses more natural resources and puts more pressure on the environment, including increasing disposal of food waste and packaging	Acceptance of these guidelines is unknown in this paper

*FBDG* food-based dietary guidelines, *FAO* food and agriculture organisation, *UPF’s* ultra-processed foods

Nine studies were found (Box 1) that: compared language in FBDGs for multiple countries (3/9 studies); described/analysed language in single country guidelines (5/9 studies); or explored the acceptability and cultural appropriateness of the Ethiopian guidelines [9].

Of the nine studies, three explored use of specific words (snacking, processed foods, and cooking) and authors reported recurring negative language where emphasis was placed on nutrients or foods, like fat or salt, that should be limited. Brito de Oliveira and colleagues evaluated culinary content of messages in FBDGs and found that only one of the 73 FBDGs available on the Food and Agriculture Organization (FAO) repository referred to cooking as a pleasurable practice [10]. Most referenced food preparation as a way to limit foods. Anastasiou et al., observed that foods-to-limit (FTL) recommendations were frequently softened with words like “moderate” [11], which are poorly understood by the public [12]. The authors note that this aligns with findings from other studies, including that of Bekele et al. [9] where consumers expressed a need for definitions of technical terms, such as ‘balanced diet’ or ‘processed foods’ [12]. They also describe an increase in use of processing-related terms over the last 20 years but report that many FBDGs do not distinguish between levels of processing. Da Silva Oliveira & Silva-Amparo note in their comparison of the 2006 and 2014 Brazilian guidelines [13], which used NOVA to classify levels of processing [14], that we need to better understand the “social and environmental consequences of ultra-processed food production”.

## Framing Concepts that Express Cultural, Economic and Environmental Reasons for Food Choices

FBDGs were developed in the mid twentieth century at a time when food was scarce, and they focused on ensuring people had enough food to avoid nutritional deficiencies. As food systems industrialised post-war, foods high in fat, sugar and salt became more available, affordable and marketed [15]. Researchers established a link between overconsumption of these nutrients and chronic diseases [11], and messaging shifted from “eat more” to “eat less” with guidelines targeting specific nutrients (eg saturated fat) rather than whole foods (eg butter) or food groups (eg fats and oils). An unintended consequence of focusing on individual nutrients may be misrepresentation of foods as less rather than more than the sum of nutrients they contain [16], and obscuring reasons, beyond health, for people’s food choices such as spending time with family and friends, cultural expression, affordability and protecting the environment.

According to the FAO, “the world cannot feed itself sustainably without listening to Indigenous Peoples”. [17]. Consequently, we framed concepts of food (Indigenous management, stewardship and enhancement of ecosystems, agro-systems and food resources), culture (intact cultures and institutions that sustain and enhance good nutrition) and nature (intact, diverse ecosystems with strong well-balanced, functioning food webs) from a chapter on global environmental challenges to the integrity of Indigenous Peoples’ food systems in a 2013 FAO publication [18]. Collectively, these concepts link to “healthy environments and healthy people with strong diversity, sustainability, safety, availability, accessibility of nutritious foods for all” [18]. We added a concept of livelihoods to acknowledge economic reasons for people’s food choices [19].

We then defined these concepts by identifying terms that capture how people express them through food and that capture food-related, cultural, economic, and environmental reasons for food choices. For example, for the concept of livelihoods, we used the following terms to describe how people balance budgets through food (budget, welfare, food storage, refrigeration, gardening, share, food programs, bulk buying) and economic reasons for why people eat (afford/affordability/affordable, availability, cost, expense, cheap, inexpensive). Suitability of the terms (i.e. in common use and appropriate in dietary guidelines) was discussed and agreed on by the two researchers. We searched for these terms in the Brazilian and Australian guidelines and extracted information on the number of times they were mentioned, where they were mentioned (eg main body, appendices, references) and on the context they were mentioned in. Lastly, we reported this information for Brazil and Australia (see Box 2) under the headings of food, culture, livelihoods and nature to compare how and where these terms were used in the guidelines. We compared the 2013 “Eat for Health Australian Dietary Guidelines” and the english language version of the 2014 “Dietary Guidelines for the Brazilian Population”. We analysed 166 pages (excluding references) of the Australian dietary guidelines. Of these, 99 pages were the main body of the guidelines (beginning of introduction to the end of “guideline 5”) and 67 pages were supplementary content, such as the preface, appendices, and glossary. We analysed 77 pages of the Brazilian guidelines after excluding the participant’s section. The body of the text (beginning of introduction to the end of “ten steps to healthy diets”) comprised of 59 pages, while the remaining 18 pages were supplementary content.

Box 2: Comparison of words used in the Brazilian and Australian dietary guidelines to describe food, culture, household economics and nature.

**Table:** 

Food—How			Food—Why		
Search terms that describe how people eat food: cereals, meats, fruit, vegetables, dairy, soy, low fat/reduced fat, lean, sugar, cuisine, condiments, dish, restaurant, cooking, breakfast, lunch, dinner, meal(s)			Search terms that describe why people eat food: taste, flavour, wholesome, fresh		
Term	Brazilian guidelines	Australian guidelines	Term	Brazilian guidelines	Australian guidelines
Cereal(s)	20 mentions (1 in supplementary content) Frequently mentioned in Ultra-processed foods section of foods to avoid “Cereal” is often used in place of “grains” (corn, rice, wheat)	158 mentions (46 in supplementary content) Says not to avoid cereals but to choose options lower in salt, fortified and wholegrain options	Taste	2 mentions “More time is also needed to follow the recommendations for the act of eating itself, to eat meals regularly, leisurely, to enjoy the pleasure of the sight, aroma, texture, and taste of food as prepared, and to share this pleasure with family, friends, or colleagues.”	7 mentions
Meat(s)	59 mentions (2 in supplementary content)	170 mentions (54 in supplementary content) Small section on how to prepare meat safely Processed meat and cancer	Flavour	9 mentions	3 mentions
Fruit(s)	88 mentions	288 mentions (139 in supplementary content)			
Vegetable(s)	96 mentions (2 in supplementary content)	318 mentions (143 in supplementary content)			
Cuisine	9 mentions	0 mentions			
Cook(ing)	Cook = 10 Cooking = 39	Cook = 10 Cooking = 25			
Meal(s)	153 mentions (9 in supplementary content)	40 mentions (14 in supplementary content)			
Lunch(es)	14 mentions	0 mentions*			
Breakfast	15 mentions	16 mentions (11 in supplementary content)			
Dinner	13 mentions	1 mention Avoid discretionary salt (…) at the dinner table			
Condiments	0 mentions	1 mention			
Lean	17 mentions	59 mentions			
Dairy	3 mentions Mentioned each time as something to limit or avoid	62 mentions (28 in supplementary content)			
Soy	5 mentions	37 mentions (16 in supplementary content)			
Dish	5 mentions	0 mentions			
Sugar	63 mentions	197 mentions (50 in supplementary content)			
Restaurant	14 mentions	1 mention			
Reduced fat	0 mentions	38 mentions (4 in supplementary content)			

*1 mention in the list of references

**Table:** 

Culture—How			Culture – Why		
Search terms that describe how people express culture through food: family, friends, community, neighbourhood			Search terms that describe cultural reasons for why people eat: culture, values, heritage, tradition(s)/traditional/traditionally, social, wellbeing		
Term	Brazilian guidelines	Australian guidelines	Term	Brazilian guidelines	Australian guidelines
Family	36 mentions (2 in annex) A lot about spending time with family, preparing food with family	36 mentions (20 in supplementary content) Mainly regarding children and breastfeeding	Culture / cultural	39 mentions 10 mentions in section about Ultra-processed foods	Culture = 3 mentions cultural(ly) = 31 mentions (13 in appendices)
Friends	13 mentions	1 mention in appendices	Values	1 mention “Cultural values”	17 mentions (10 in supplementary content) Other mentions of value were with reference to nutritional rather than cultural values
Community	15 mentions (1 in supplementary content) Mainly in “understanding & overcoming obstacles” section	61 mentions (45 in supplementary content) Mainly about community support for breastfeeding	Heritage	3 mentions Cultural and culinary heritage	0 mentions
Neighbourhood	3 mentions	0 mentions*	Tradition(s) / traditional / traditionally	17 mentions All in main text	26 mentions (19 in appendices) Mainly “traditional”, mix of “traditional foods” and “traditional(ly)” as in “historically, or back in the day” Mainly in section for Aboriginal and Torres Strait Islander Australians
			Social	84 mentions (22 in supplementary content)	71 mentions (15 in supplementary content)
			Wellbeing	1 mention	22 mentions Often combined with health as in “health and wellbeing”

* 3 mentions in the list of references

**Table:** 

Livelihood- How			Livelihood—Why		
Search terms that describe how people balance budgets through food: budget, welfare, food storage, refrigeration, gardening, share, food programs, bulk buying			Search terms that describe economic reasons for why people eat: afford/affordability/affordable, availability, cost, expense, cheap, inexpensive		
Term	Brazilian guidelines	Australian guidelines	Search term	Brazilian guidelines	Australian guidelines
Budget / budgetary	2 mentions	4 mentions*	Affordability/ affordable/afford	8 mentions Mainly gives examples for making meals affordable	11 mentions 3 mentions that affordability may be a barrier to compliance with recommendations
Welfare (financial)	1 mention	3 mentions ^	Availability / available	8 mentions All about how ultra-processed foods are available everywhere but minimally processed foods are not	98 mentions
Store (Food storage)	6 mentions	21 mentions	Cost	25 mentions	69 mentions
Refrigeration	5 mentions	8 mentions	Expense	1 mention	0 mentions
Gardening	4 mentions 1 mention of community gardens	4 mentions (one related to using gardening as a form of exercise)	Cheap	9 mentions	0 mentions
Share	29 mentions Related to sharing meals, prep/cooking tasks, ideas, cooking skills/knowledge, pleasure, and responsibility	0 mentions	Inexpensive	1 mention	4 mentions
Program (food programs)	6 mentions—all about how these guidelines are designed to help improve food programs	27 mentions (2 in supplementary content) ^#^	Food security	(2 mentions in supplementary content)	11 mentions “The cost of nutritious foods in these [remote] areas is also over 30% higher than in major cities and may impact on food security”
Bulk (bulk buying)	4 mentions related to buying food in bulk	0 mentions	Access/ accessible/ accessibility	17 mentions	47 mentions (19 in supplementary content) ^&^

* 1 mention in the list of references, ^ 27 mentions in the list of references, # 19 mentions in reference list, ^&^ 11 mentions in reference list

**Table:** 

Nature—How			Nature—Why		
Search terms that describe how people acknowledge or preserve nature through food: garden/ing, season/ally, vegan, vegetarian			Search terms that describe environmental reasons for why people eat: environment/environmental/environmentally, organic, land, biodiversity, local, sustainable, sustainability, conservation, climate		
Term	Brazilian guidelines	Australian guidelines	Term	Brazilian guidelines	Australian guidelines
Season(al) / seasonally	27 mentions	5 mentions	Environment /environmental/environmentally	60 mentions 35 mentions regarding planetary health in first half of guidelines Often mentioned together with “social” as in environmental and social impacts of food 6 mentions in ultra-processed foods section 8 mentions of physical environment (where you eat)	126 mentions (78 mentioned in supplementary content) Mainly related to school/work environment, social environment “Discussion of dietary patterns and the environment is included in Appendix G.” mentioned 6 times
Garden/gardening	4 mentions Growing your own food Community gardens	4 mentions—2 in appendices Gardening for Physical Activity Use compost bins in garden	Organic	16 mentions (3 in supplementary content)	7 mentions
Vegan/vegetarian	0 mentions	19 mentions Inclusive of those who follow a vegan diet	Land	3 mentions “Industrial systems demand more land” Minimally processed foods contribute to distribution of productive lands	0 mentions
			Biodiversity	11 mentions	10 mentions
			Local	17 mentions	7 mentions (6 in supplementary content) “In rural and remote areas a wide variety of fresh foods may not be locally available or may be expensive. Available traditional foods can be a nutritious alternative”
			Sustainable/sustainability	23 mentions (5 in supplementary content) “Sustainable food systems” mentioned 11 times	66 mentions (42 in supplementary content)*
			Conservation	3 mentions Soil, forests, how to conserve food	2 mentions 1 in appendix 1 in ref list
			Climate (nothing about climate change)	2 mentions How much water one needs depends on the climate	4 mentions (1 in supplementary content) ^ “In general, self-regulation of excess water consumption occurs in healthy people in temperate climates.”

* 21 in list of references, ^ 2 in list of references

## Representations of Food, Culture, Livelihoods and Nature in the Australian and Brazilian Dietary Guidelines

### Food

Compared to the Australian guidelines, the Brazilian guidelines used fewer words to describe individual food groups and more to describe meals and mealtimes. For example, the word “meal(s)” was mentioned 153 times in the main body of the Brazilian guidelines, whereas it was mentioned 40 times in the Australian guidelines. On the other hand, the words cereals, meats, fruits, vegetables and dairy were mentioned 158 times, 170 times, 288 times, 318 times and 62 times in the Australian guidelines respectively compared to 20 times, 59 times, 88 times, 96 times, and 3 times in the Brazilian guidelines. Sugar was mentioned 197 times in the Australian guidelines compared to 63 times in the Brazilian guidelines. The relative absence of individual food groups in the Brazilian guidelines compared to the Australian guidelines may be because of the use of the NOVA food classification system which captures food groups under four processing categories (unprocessed/minimally processed foods, processed culinary ingredients, processed foods, ultra-processed foods) [14]. Terms capturing why people eat were not common in either guideline with taste and flavour being the only two searched for terms that were mentioned, both less that 10 times. That said, the Brazilian guidelines recommended that people give “more time … for the act of eating itself” [7].

### Culture

Words that expressed culture through food were common in both countries’ guidelines. The words “family”, “community”, and “neighbourhood”, were mentioned 54 times in the Brazilian guidelines and 97 times in the Australian guidelines. In the Australian guidelines, “family” and “community” were almost exclusively used with reference to breastfeeding. Terms that captured cultural reasons for why people eat were also common with “culture/urally”, “values”, “heritage”, “tradition”, “social” and “wellbeing” mentioned 145 and 169 times in the Brazilian and Australian guidelines respectively. The terms “culture/urally”, “tradition” and “social” were mentioned as much in supplementary content of the Australian guidelines as they were in the main body of the guidelines and “tradition” was mainly mentioned in a section for Aboriginal and Torres Strait Islander Australians. The context these words were used in was different however. In the Brazilian guidelines, they were most frequently used in the context of spending time with family, sharing responsibilities, eating in community with others, and preserving aspects of traditional Brazilian culinary cuisine and culture. Australian guidelines lacked encouragement to eat meals in community with family and friends and to share household responsibilities associated with gathering and preparing food. In keeping with this observation, “friends” was mentioned 13 times and “restaurants” was mentioned 14 times in the Brazilian guidelines whereas, there was only one mention of each of these words in the Australian guidelines.

### Livelihoods

In both guidelines, there were fewer mentions of words related to household economics than words related to the other concepts explored. For example, budget was mentioned 2 times in Brazilian guidelines and 4 times in Australian guidelines. Affordability, and derivations, was mentioned 8 times in the Brazilian guidelines, which were mainly examples of how to make meals affordable, and 11 times in the Australian guidelines (with 3 noting that affordability may be a barrier to compliance with the recommendations). Terms capturing economic reasons for why people eat were more prominent in the Australian guidelines, with cost mentioned 69 times (compared to 25 in the Brazilian guidelines) and availability mentioned 98 times (compared to 8 times in the Brazilian guidelines and, those mentions were in relation to availability of ultra-processed foods). Interestingly, “share”, as in sharing meals or food preparation tasks, was mentioned 29 times and “cheap” was mentioned 9 times in the Brazilian guidelines but neither word was used in the Australian guidelines.

### Nature

“Season/al” was mentioned 27 times in the Brazilian guidelines but only 5 times in the Australian guidelines. “Garden/ing” was mentioned 4 times in both guidelines. “Vegan/vegetarian” was not mentioned in the Brazilian guidelines but had 19 mentions in the Australian guidelines. With respect to terms capturing environmental reasons for why people eat, “environment”, and derivations, was mentioned 60 times in the Brazilian guidelines, and more than half of these mentions referred to planetary health. “Environment”, and derivations, was mentioned 126 times in the Australian guidelines although mostly to distinguish food settings (eg school, work, or social environments). “Organic” was mentioned twice as often (16 mentions vs 7 mentions) in the Brazilian guidelines and “land” 3 times compared to zero mentions in the Australian guidelines. “Biodiversity” was mentioned ~ 10 times in both guidelines while “local” was more common in the Brazilian guidelines (17 mentions vs 7 mentions). “Sustainable/sustainability” was more common in the Australian guidelines (66 mentions vs 23 mentions). However, not all mentions in the Australian guidelines described environmental sustainability and the majority (42) were in supplementary content rather than in the main body and, half of these (21) were in the reference list. The Brazilian guidelines mentioned “sustainable food systems” 11 times. Climate was mentioned (2 times in the Brazilian guidelines, 4 times in the Australian guidelines) in relation to water consumption. There were no mentions of climate change in either set of guidelines, possibly because they were published at a time when awareness of the impact of food systems on climate was higher among environmental compared to nutrition scientists [20].

### Strengths and Limitations

A strength of this review is its focus on concepts, informed by Indigenous food systems, and associated terms, that capture reasons why people make food choices. If these terms became more common in dietary guidelines, the populations for whom they are designed may benefit more from them. The main limitations are that the review was not systematic and the terms we identified to capture the concepts were not exhaustive and are likely influenced by the researcher’s health promotion bias.

## Conclusion

Some but not a lot of attention has been paid to language use in dietary guidelines. This brief review suggests language in FBDGs should be unambiguous for consumers and evolve with national nutrition priorities. For example, considering global climate change and cost of living crises, we recommend the next iterations of the Australian and Brazilian guidelines give more consideration to environmental and economic reasons for food choices and use associated language. A notable difference between Australian and Brazilian FBDGs was that Australia centralised individual food groups and individuals, whereas Brazil placed people in an ecosystem. Dietary guidelines in these countries would benefit from including more words that speak to how food is prepared and eaten, to expressions of culture and community, and to strategies people use for budgeting as well as enhancing and protecting planetary health.

## Key References

- Brito de Oliveira MC, Martins C, De Castro IRR. The (scarce and circumscribed) culinary content in food-based dietary guidelines around the world: 1991–2021. *Public Health Nutrition* 2022 25(12), 3559–3567. 10.1017/s1368980022001938.
  - This review of global food-based dietary guidelines (73 that were available in English) was the first to analyse culinary content in key messages of food-based dietary guidelines. The review identified that cooking, an important influence on food choice, was not well promoted in guidelines.
- Da Silva Oliveira MS, Silva-Amparo L. (2017). Food-based dietary guidelines: a comparative analysis between the Dietary Guidelines for the Brazilian Population 2006 and 2014. Public Health Nutrition, 21(1), 210–217. 10.1017/s1368980017000428
  - This article provides the results of a discourse analysis, comparing the language used in the 2006 Brazilian dietary guidelines to the 2014 dietary guidelines. It is one of the first to use the power of discourse analysis for exploring whether guidelines achieve intended goals.
- Koios D, Machado PP, Lacy-Nichols J. Representations of Ultra-Processed Foods: A Global Analysis of How Dietary Guidelines Refer to Levels of Food Processing. *International Journal of Health Policy and Management* 2022. 10.34172/ijhpm.2022.6443
  - This review of 106 dietary guidelines, using a qualitative content analysis, found that most do not adequately speak to the level of food processing.
- Food and Agriculture Organisation and Alliance of Bioversity International and International Centre for Tropical Agriculture. Indigenous Peoples’ food systems: Insights on sustainability and resilience in the front line of climate change. Rome. 2021 10.4060/cb5131en
  - This publication is the third in a series from the Food and Agriculture Organisation that explore Indigenous Food Systems. Collectively, using case studies from around the world, they provide learnings on how to achieve sustainable and efficient food systems that better support nutrition and health.

## Data Availability

No datasets were generated or analysed during the current study.
